# Multi-task learning sparse group lasso: a method for quantifying antigenicity of influenza A(H1N1) virus using mutations and variations in glycosylation of Hemagglutinin

**DOI:** 10.1186/s12859-020-3527-5

**Published:** 2020-05-11

**Authors:** Lei Li, Deborah Chang, Lei Han, Xiaojian Zhang, Joseph Zaia, Xiu-Feng Wan

**Affiliations:** 1 Department of Basic Sciences, College of Veterinary Medicine, Mississippi State University, Mississippi State, MS USA; 2 Center for Biomedical Mass Spectrometry, Department of Biochemistry, Boston University School of Medicine, Boston, MA USA; 3 Tencent AI Lab, Shenzhen, China; 4 Department of Molecular Microbiology and Immunology, School of Medicine, University of Missouri, Columbia, MO USA; 5 MU Center for Research on Influenza Systems Biology (CRISB), University of Missouri, Columbia, MO USA; 6 Bond Life Sciences Center, University of Missouri, Columbia, MO USA; 7 Department of Electrical Engineering & Computer Science, College of Engineering, University of Missouri, Columbia, MO USA; 8 MU Institute for Data Science and Informatics, University of Missouri, Columbia, MO USA

**Keywords:** Influenza virus, H1N1, Multi-task learning, Sparse learning, Group lasso, LASSO, MTL-SGL, *N*-linked glycosylation, Vaccine strain selection, Antigenic drift

## Abstract

**Background:**

In addition to causing the pandemic influenza outbreaks of 1918 and 2009, subtype H1N1 influenza A viruses (IAVs) have caused seasonal epidemics since 1977. Antigenic property of influenza viruses are determined by both protein sequence and *N*-linked glycosylation of influenza glycoproteins, especially hemagglutinin (HA). The currently available computational methods are only considered features in protein sequence but not *N*-linked glycosylation.

**Results:**

A multi-task learning sparse group least absolute shrinkage and selection operator (LASSO) (MTL-SGL) regression method was developed and applied to derive two types of predominant features including protein sequence and *N*-linked glycosylation in hemagglutinin (HA) affecting variations in serologic data for human and swine H1N1 IAVs. Results suggested that mutations and changes in *N*-linked glycosylation sites are associated with the rise of antigenic variants of H1N1 IAVs. Furthermore, the implicated mutations are predominantly located at five reported antibody-binding sites, and within or close to the HA receptor binding site. All of the three *N*-linked glycosylation sites (i.e. sequons NCSV at HA 54, NHTV at HA 125, and NLSK at HA 160) identified by MTL-SGL to determine antigenic changes were experimentally validated in the H1N1 antigenic variants using mass spectrometry analyses. Compared with conventional sparse learning methods, MTL-SGL achieved a lower prediction error and higher accuracy, indicating that grouped features and MTL in the MTL-SGL method are not only able to handle serologic data generated from multiple reagents, supplies, and protocols, but also perform better in genetic sequence-based antigenic quantification.

**Conclusions:**

In summary, the results of this study suggest that mutations and variations in *N*-glycosylation in HA caused antigenic variations in H1N1 IAVs and that the sequence-based antigenicity predictive model will be useful in understanding antigenic evolution of IAVs.

## Background

The subtype of an influenza A virus (IAV) is determined based on the subtypes of its virus surface glycoproteins, hemagglutinin (HA) and neuraminidase (NA). A total of 18 HA and 11 NA subtypes have been reported for IAVs [1]. Among hundreds of combinations of HA and NA subtypes, H1N1 is one of only a few causing significant burdens to public health. Two of four documented influenza pandemics (in 1918 and 2009) were caused by subtype H1N1 IAVs, and the 1918 resulting in > 40 million human deaths worldwide [2–5]. In addition, H1N1 IAVs have been a predominant cause of seasonal influenza outbreaks between 1918 to 1957 and since 1977. Strains of the 1977 influenza A(H1N1) virus were responsible for outbreaks during the 1977–78 to 2008–09 seasons, but in 2009, influenza A(H1N1)pdm09 virus took over as the new seasonal influenza virus. Genetic characterization suggested that the 1918 A(H1N1) pandemic [hereafter referred to as A(H1N1)pdm1918] virus was the precursor to the seasonal 1977 A(H1N1) [hereafter referred to as A(H1N1)season1977] virus and the A(H1N1)pdm09 virus [6]. Sequence analyses showed numerous mutations in the HA of these A(H1N1)season1977 and A(H1N1)pdm09 viruses, including mutations in antibody binding sites and glycosylation sites [7]. Serologic characterization suggested that A(H1N1)pdm1918 has a low level of cross-reactivity with A(H1N1)pdm09 and that A(H1N1)season1977 and A(H1N1)pdm09 do not cross-react with each other [8–11].

Among influenza viruses, antigenic drift is caused by gradual changes in the virus surface glycoproteins, HA and/or NA, whereas antigenic shift is caused by reassortment with HA and/or NA that is antigenically distinct from that in strains endemic to humans. Antigenic drift is often seen in seasonal IAVs [e.g., A(H1N1)season1977], whereas antigenic shift can lead to emergence of a pandemic virus [e.g., A(H1N1)pdm09] [12–14]. Antigenic changes by drift or shift allow IAVs to evade the accumulating herd immunity from prior influenza infections and/or vaccination, and thus present great challenges in influenza vaccine strain selection. During 1977–2017, such changes resulted in 12 updates for the human influenza vaccine [15].

Recent advances in sequencing technology have allowed us to identify genetic changes rapidly in influenza genomes, and genome sequencing has become one of the routine procedures in the influenza surveillance program [16–19]. Thus, a genome-based vaccine strain selection strategy would be ideal. To develop an effective in silico model to quantify antigenic distances between IAVs solely by using sequences, it is essential that we understand the key structural features determining influenza virus antigenicity. Previous studies have suggested that antigenicity is affected predominantly by changes in the antibody binding sites (i.e., ~ 100 residues) mostly located in the head structure of the HA protein [20–22]. Only one or a few of these antigenicity-associated sites change frequently during antigenic drift events [23–26]. Mutations at these sites can lead to changes in peptidic epitope structures. Those changes can cause deviations in immunologic responses in serologic assays through modification of biophysical properties of amino acids or modification of glycosylation patterns [27]. For H1N1 IAVs, correlation analyses, through linear mixed-effects modeling, between ferret sera–derived hemagglutination inhibition (HI) data and HA protein sequences for A(H1N1)season1977 suggested that a few mutations in HA sequences affected antigenic changes among A(H1N1)season1977 from 1999 to 2009 [28]. In addition, *N*-linked glycosylation (hereafter referred to as *N*-glycosylation) of HA was considered to be associated with antigenic variations between A(H1N1)pdm09 and A(H1N1)season1977 [29]. Nevertheless, molecular determinants for antigenic changes in H1N1 IAVs are still not fully understood, especially the roles of *N*-glycosylation.

We formulated the study of antigenicity as a multi-task sparse learning problem with the aim to identify gene sequence, proteome, and site-specific *N*-glycosylation as antigenicity determinants. We developed a multi-task learning sparse group least absolute shrinkage and selection operator (LASSO) (MTL-SGL) machine-learning model to assess antigenic changes in human, swine, and avian H1N1 IAVs. All of three *N*-linked glycosylation sites identified by MTL-SGL to determine antigenic changes were experimentally validated using glycoproteomics. Based on these three features, we developed a sequence-based model and used it to illustrate the antigenic evolution of H1N1 IAVs.

## Methods

### Data

Serologic data for H1N1 viruses were collected from data described elsewhere [11, 30, 31], including 2030 HI titers generated between 153 viruses and 97 serum samples (Table S1). A total of 13,591 non-identical H1 protein sequences were obtained from Influenza Virus Resource [32], Influenza Research Database [33], and GISAID [34]. Sequences and serologic data can be accessed at https://github.com/InfluenzaSystemsBiology/MTL-SGL.

### MTL-SGL regression model

The overall objective of this study is to identify mutations and changes in *N*-linked glycosylation sites in HA proteins being associated with antigenic changes of subtype H1N1 IAVs and further to develop a quantitative function for quantifying antigenic distances giving protein sequences of HA proteins. In this study, we formulated this problem into a multi-task sparse learning problem (see the details in problem formulation) by the facts that previous studies suggested that sparse learning algorithm was effective in identifying antigenicity associated features in protein sequences from multiple subtypes of IAVs, including H5N1 [35], H3N2 [36–39], and H1N1 [28], and that multi-task formulation can overcome the challenges in data integration for influenza serological data [38]. To make our model be flexible to integrate multiple types of features, group Lasso is further introduced into MTL-SPG so that the model can learning simultaneously two types of features affecting influenza virus antigenicity (i.e. sequence and *N*-glycosylation).

The advantages of sparse learning over other conventional machine learning approaches are that its efficiency and generalizability generate accurate models using a small number of non-zero elements. Sparse learning also takes advantage of the sparsity of predominant features in influenza proteins. This is important because high dimensional features can be redundant and noisy, resulting in poor generalization performance [40]. The parse learning approach addresses the redundancy and noise levels present in replication efficiency data. Thus, we expect the sparse learning method will increase performance in feature selection and facilitate data interpretation. In addition, Lasso can be effective in handling small data size and this is fit for our application.

#### Problem formulation

**Fig. 1:**
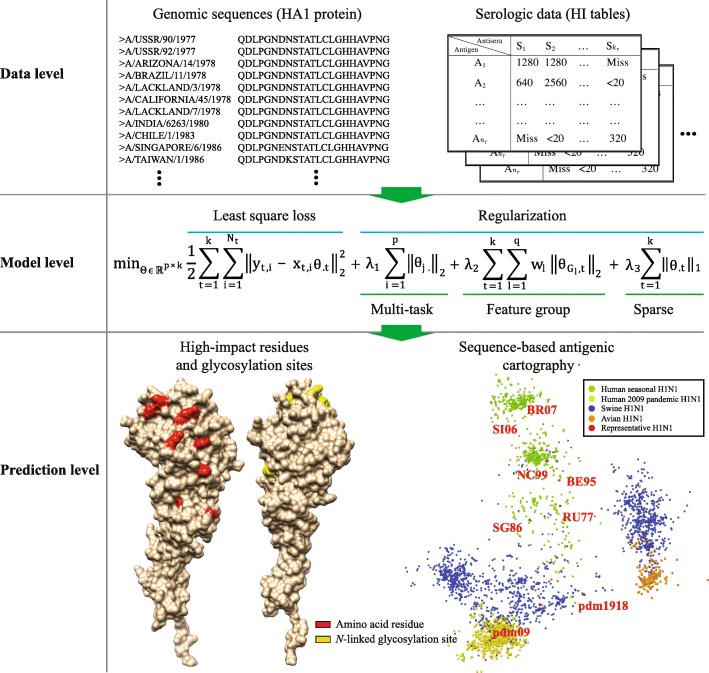
Workflow of multi-task learning sparse group LASSO (MTL-SGL). The framework was composed of three levels: data, model, and prediction levels. To avoid bias from sample sizes, training data were divided into several subsets according to their data source, data type, and antigenic clusters. The pairwise genetic difference matrix and pairwise antigenic distance vector were generated from hemagglutination inhibition (HI) assay data and hemagglutinin subunit HA1 sequence data in the data level for each dataset. The MTL-SGL model selected antigenicity-associated features by analyzing correlations between genotype and phenotype by minimizing an objective function. In the model level, each selected feature is given a numeric weight as its impact. Antigenic distances among new viruses can be inferred from their HA1 sequences in the prediction level by weights from the model level. Sequence-based maps are generated by using a multidimensional scaling algorithm from pairwise antigenic distances inferred from HA1 sequences. Min, minimum; miss, missing. Antigenic cluster: BE95, A/Beijing/262/1995(H1N1)-like virus; BR07, A/Beijing/262/1995(H1N1)-like virus; NC99, A/New Caledonia/20/1999(H1N1)-like virus; pdm09, A(H1N1)pdm09-like virus; pdm1918, 1918 pandemic H1N1-like virus; RU77, A/USSR/90/1977(H1N1)-like virus; SG86, A/Singapore/6/1986(H1N1)-like virus; SI06, A/Solomon Islands/3/2006(H1N1)-like virus

Under the hypothesis that a small set of features encoded in HA would determine antigenic profiles of IAVs, the MTL-SGL model integrates multiple groups of features and assigned each feature in each group a numeric weight indicating the importance of each feature on antigenicity determination. The larger the weight, the more important a corresponding feature. Serologic (i.e., HI) data were used to generate a phenotype distance matrix, and key features were identified to reproduce the genotype difference matrix (Fig. 1). Mathematically, MTL-SGL model can be expressed as

1 $$ {\min}_{\Theta}\kern0.75em \mathrm{L}\left(\mathrm{X},\mathrm{Y},\Theta \right)+\uplambda \mathrm{R}\left(\Theta \right), $$

where L(X, Y, Θ) is the square loss function and R(Θ) is the regularizer.

2 $$ \mathrm{L}\left(\mathrm{X},\mathrm{Y},\Theta \right)=\frac{1}{2}{\left\Vert \mathrm{Y}-\mathrm{X}\Theta \right\Vert}_{\mathrm{F}}^2=\frac{1}{2}\sum \limits_{\mathrm{t}=1}^{\mathrm{k}}\sum \limits_{\mathrm{i}=1}^{{\mathrm{N}}_{\mathrm{t}}}{\left\Vert {\mathrm{y}}_{\mathrm{t},\mathrm{i}}-{\mathrm{x}}_{\mathrm{t},\mathrm{i}}{\uptheta}_{.\mathrm{t}}\right\Vert}_2^2\kern1em $$

3 $$ \uplambda \mathrm{R}\left(\Theta \right)={\uplambda}_1{\mathrm{R}}_1\left(\Theta \right)+{\uplambda}_2{\mathrm{R}}_2\left(\Theta \right)+{\uplambda}_3{\mathrm{R}}_3\left(\Theta \right) $$

where t is the subscript for the t-th task, k is the total number of tasks, i is the subscript for the i-th sample in each task, N_t_ is the number of samples in the t-th task and θ._t_ is the weight vector for the t-th task. R(Θ) is composed of three components:

4 $$ {\mathrm{R}}_1\left(\Theta \right)={\left\Vert \Theta \right\Vert}_{2,1}=\sum \limits_{\mathrm{j}=1}^{\mathrm{p}}{\left\Vert {\uptheta}_{\mathrm{j}.}\right\Vert}_2 $$

5 $$ {\mathrm{R}}_2\left(\Theta \right)=\kern0.5em {\left\Vert {\Theta}_{\mathrm{G}}\right\Vert}_{2,1}=\sum \limits_{\mathrm{t}=1}^{\mathrm{k}}\sum \limits_{\mathrm{l}=1}^{\mathrm{q}}{\mathrm{w}}_{\mathrm{l}}{\left\Vert {\uptheta}_{{\mathrm{G}}_{\mathrm{l}},\mathrm{t}}\right\Vert}_2 $$

6 $$ {\mathrm{R}}_3\left(\Theta \right)={\left\Vert \Theta \right\Vert}_1=\sum \limits_{\mathrm{t}=1}^{\mathrm{k}}{\left\Vert {\uptheta}_{.\mathrm{t}}\right\Vert}_1 $$

where j is the subscript for the feature, p is the total number of features, G_l_ denotes feature groups, q is the number of feature groups, ${\mathrm{w}}_{\mathrm{l}}=\sqrt{{\mathrm{m}}_{\mathrm{l}}}$ is the weight of feature group G_l_, θ_j._ denotes the weight for the j-th feature across different tasks, and ${\uptheta}_{{\mathrm{G}}_{\mathrm{l}},\mathrm{t}}$ denotes the weight for feature group G_l_ of the t-th task. Then, the objective function will be

$$ {\min}_{\Theta \in {\mathbb{R}}^{\mathrm{p}\times \mathrm{k}}}\kern0.75em \mathrm{L}\left(\mathrm{X},\mathrm{Y},\Theta \right)+\uplambda \mathrm{R}\left(\Theta \right) $$

which equals to

7 $$ {\min}_{\Theta \in {\mathbb{R}}^{\mathrm{p}\times \mathrm{k}}}\kern0.75em \mathrm{L}\left(\mathrm{X},\mathrm{Y},\Theta \right)+{\uplambda}_1{\mathrm{R}}_1\left(\Theta \right)+{\uplambda}_2{\mathrm{R}}_2\left(\Theta \right)+{\uplambda}_3{\mathrm{R}}_3\left(\Theta \right) $$

or

8 $$ {\min}_{\Theta \in {\mathbb{R}}^{\mathrm{p}\times \mathrm{k}}}\frac{1}{2}\sum \limits_{\mathrm{t}=1}^{\mathrm{k}}\sum \limits_{\mathrm{i}=1}^{{\mathrm{N}}_{\mathrm{t}}}{\left\Vert {\mathrm{y}}_{\mathrm{t},\mathrm{i}}-{\mathrm{x}}_{\mathrm{t},\mathrm{i}}{\uptheta}_{.\mathrm{t}}\right\Vert}_2^2+{\uplambda}_1\ \sum \limits_{\mathrm{j}=1}^{\mathrm{p}}{\left\Vert {\uptheta}_{\mathrm{j}.}\right\Vert}_2+{\uplambda}_2\sum \limits_{\mathrm{t}=1}^{\mathrm{k}}\sum \limits_{\mathrm{l}=1}^{\mathrm{q}}{\mathrm{w}}_{\mathrm{l}}{\left\Vert {\uptheta}_{{\mathrm{G}}_{\mathrm{l}},\mathrm{t}}\right\Vert}_2+{\uplambda}_3\sum \limits_{\mathrm{t}=1}^{\mathrm{k}}{\left\Vert {\uptheta}_{.\mathrm{t}}\right\Vert}_1 $$

where λ_1_, λ_2_, and λ_3_ are regularization parameters. Figure 2 illustrates the MTL-SGL model.

**Fig. 2:**
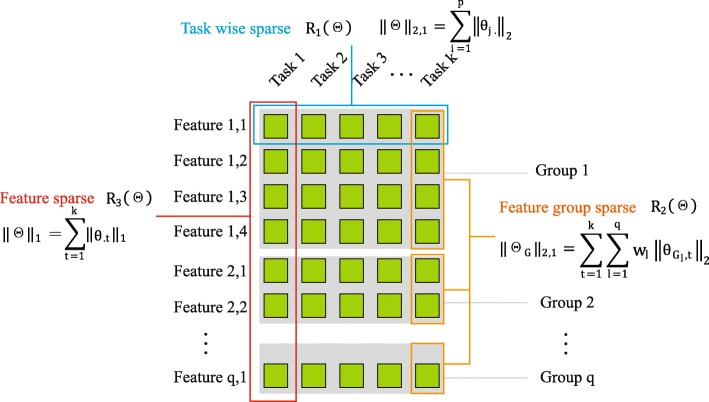
The MTL-SGL model. Blocks indicate individual features across tasks. Gray shadings indicate feature groups. Three regularizers, R_1_(Θ), R_2_(Θ), and R_3_(Θ), are indicated by blue, orange, and red boxes, respectively

#### Solving MTL-SGL model using the alternating direction method of multipliers algorithm

We apply the alternating direction method of multipliers (ADMM) algorithm [41] to solve the model in eq. (8). We assume

$$ {\mathrm{R}}_{\uplambda_1,{\uplambda}_2,{\uplambda}_3}\left(\Theta \right)={\uplambda}_1{\left\Vert \Theta \right\Vert}_{2,1}+{\uplambda}_2{\left\Vert {\Theta}_G\right\Vert}_{2,1}+{\uplambda}_3{\left\Vert \Theta \right\Vert}_1, $$

Then the objective function is equivalent to

9 $$ {\min}_{\Theta \in {\mathbb{R}}^{\mathrm{p}\times \mathrm{k}}}\kern0.75em \frac{1}{2}{\left\Vert \mathrm{Y}-\mathrm{X}\Theta \right\Vert}_{\mathrm{F}}^2+{\mathrm{R}}_{\uplambda_1,{\uplambda}_2,{\uplambda}_3}\left(\mathrm{Q}\right)\kern1.25em \mathrm{subject}\ \mathrm{to}\ \Theta -\mathrm{Q}=0 $$

where Q is a slack variable.

The augmented Lagrangian function is

10 $$ {\mathrm{L}}_{\uprho}\left(\Theta, \mathrm{Q},\mathrm{U}\right)=\frac{1}{2}{\left\Vert \mathrm{Y}-\mathrm{X}\Theta \right\Vert}_{\mathrm{F}}^2+{\mathrm{R}}_{\uplambda_1,{\uplambda}_2,{\uplambda}_3}\left(\mathrm{Q}\right)+\mathrm{Tr}\left({\mathrm{U}}^{\mathrm{T}}\left(\Theta -\mathrm{Q}\right)\right)+\frac{\uprho}{2}{\left\Vert \Theta -\mathrm{Q}\right\Vert}^2 $$

where U is the augmented Lagrangian multiplier and ρ is a parameter that control the rate of convergence (step size); the initial value of ρ was set to 1.5.

As shown in the following algorithm box, in each iteration, we update Θ, Q, and U separately by fixing another two according to ADMM. The steps of updating Q follow the technique used in [42, 43].

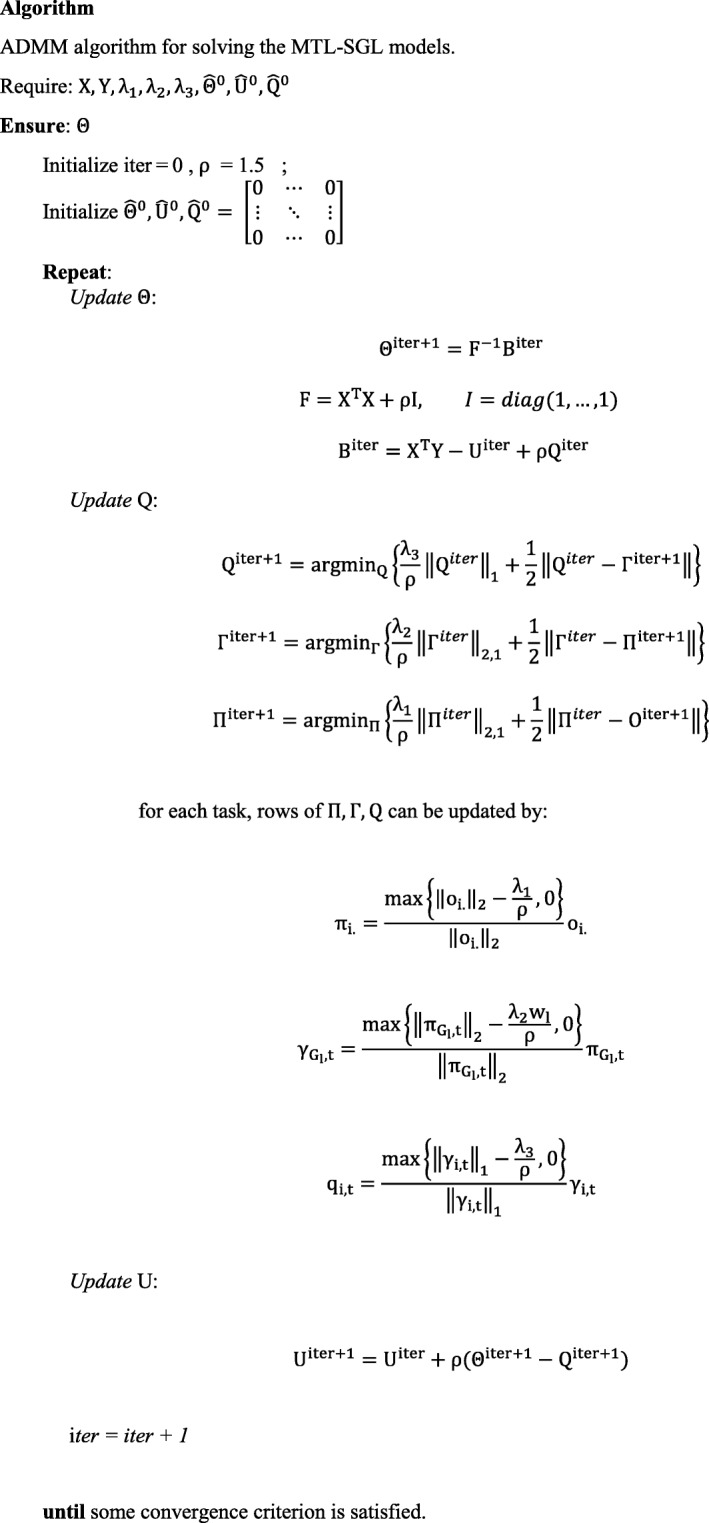



#### Defining features

In this study, two key groups of features (i.e., mutation and *N*-glycosylation) were identified and used to quantify influenza antigenicity. Of note, *O*-glycosylation has not been detected in the HA of IAVs, and thus is not considered as a feature in our model [44]. The genetic feature space (genotype data) is defined as

11 $$ {\mathrm{F}}_{1:\mathrm{p}+\mathrm{q}}^{\left(\mathrm{N},\mathrm{M}\right)}=\left\{{\mathrm{N}}_1,{\mathrm{N}}_2,\dots, {\mathrm{N}}_{\mathrm{p}}\right\}\cup \left\{{\mathrm{M}}_1,{\mathrm{M}}_2,\dots, {\mathrm{M}}_{\mathrm{q}}\right\}, $$

where {N_1_, N_2_, …, N_p_} denotes changes associated with *N*-glycosylation and {M_1_, M_2_, …, M_q_} is the set of mutations being associated without *N*-glycosylation. These two groups of features were assumed to be non-redundant. To consider the biochemical properties of amino acids, we adopted the score of pattern-induced multi-sequence alignment (PIMA) into the regularization function for M, as described elsewhere [37, 45, 46]; PIMA assigned 20 amino acids into 9 groups, and gave a different numerical coding for different mutations [46]. For *N*-glycosylation, the binary scoring schema was used as described elsewhere [35]. PIMA coding and binary coding achieved similar performance on our dataset (data not shown); thus, for consistency, a binary coding schema was used for all features.

*N*-glycosylation sites at HA protein sequences were predicted by NetNGlyc 1.0 Server (http://www.cbs.dtu.dk/services/NetNGlyc/). The evolution pattern of *N*-glycosylation sites on HA protein of H1N1 viruses was reported before; in this study, for all predicted potential *N*-glycosylation sites, only non-conserved ones were included in the machine learning model, as described elsewhere [7]. The non-conserved *N*-glycosylation sites at HA of A/Solomon Islands/03/2006(H1N1) (PDB accession number 5UG0) were further conserved using NGlycPred, which is a software predicting *N*-linked glycosylation sites incorporating structural information [47]. In addition, *N*-glycosylation sites, all amino acid residues, with a variant rate > 10%, will be considered as non-conserved sites and included in the machine learning model.

#### Sequence-based antigenic quantification model

Given the sequences of a pair of viruses, i and j, a scoring function is proposed to predict the antigenic distance between them. Suppose virus i and j are from clusters *C*_*i*_ and *C*_*j*_, then, we define our prediction model as

12 $$ \hat{\mathrm{y}}=\mathrm{x}\left(\upmu {\mathrm{w}}^{\mathrm{global}}+\frac{1-\upmu}{2}\left({\mathrm{w}}_{C_i}^{\mathrm{local}}+{\mathrm{w}}_{C_j}^{\mathrm{local}}\right)\right), $$

where x is the genetic distance vector based on the HA sequences; $\hat{\mathrm{y}}$ is the predicted antigenic distance between the two viruses; w^global^ is the global weight representing the average of weights across different tasks; and $\kern0.50em {\mathrm{w}}_{C_i}^{\mathrm{local}}$ and ${\mathrm{w}}_{C_j}^{\mathrm{local}}$ indicate the weights or the specific virus i and j in each individual task. μ is set to 0.4 to balance the global and local weights. For some rare cases in which a cluster of virus i and/or j is missing or difficult to determine, μ was set to 1 and we will predict only global weights; μ was also set to 1 for large-scale predictions across H1N1 IAVs from different antigenic clusters and/or different hosts.

#### Defining data dependent multiple tasks and multi-task low-rank matrix completion

In this study, a total of five individual tasks were designed from three datasets. Specifically, datasets 2 and 3 were each designed as individual tasks, and the data for A(H1N1)season1977 viruses from 1977 to 2009 (i.e., dataset 1) had a banded structure similar to that for the data for H3N2 seasonal influenza viruses [48]. If we arrange antigens and antibodies in an HI matrix according to time, most of the high reactors appear very close to the diagonal zone, whereas the low reactors and the missing values appear far away from the diagonal zone [48]. A low-rank matrix completion method successfully overcame this band structure specific challenge by giving an approximate estimation to the low reactors and missing values. Our prior studies suggested that multi-task matrix completion further simplified the data analyses and improved prediction performance, as described in Han et al. from whom we adapted a multi-task low-rank matrix completion platform by dividing dataset 1 into multiple tasks. Specifically, the following protocol was implemented: 1) construct an antigenic map based on the HI matrix derived from low rank matrix completion; 2) identify antigenic clusters by using the spectral clustering method; 3) define antigenic drift for neighboring antigenic clusters; 4) define each antigenic drift event as an individual task; and 5) perform matrix completion for each task individually and then generate antigenic distances.

#### Parameter tuning, performance evaluation, and bootstrapping analyses

The regularization parameters in the MTL-SGL model were tuned based on the root mean square error (RMSE) (Supplementary Information). The MTL-SGL model were compared with two MTL models (*ℓ*_1, 2_ MTL and *ℓ*_1, ∞_ MTL) and two single task models (LASSO and SGL) (Table 1) (also in Supplementary Information). In addition, to assess the confidences for the features to be selected by MTL-SGL, 100-fold independent bootstrapping analyses were performed as described elsewhere. In brief, we selected all features with a high bootstrap value (bootstrap value cutoff was set to 80) from 100 independent runs.

**Table 1:** Performance evaluation of the multi-task learning (MTL) methods (including the MTL-SGL, *ℓ*_1, 2_ MTL and *ℓ*_1, ∞_ MTL), and the single task learning methods (including Lasso regression and sparse group lasso regression). The rooted mean square error (RMSE), Average accuracy (Acc.), Average sensitivity (Sen.), Average specificity (Spe.), Area Under Receiver Operating Characteristic Curves (AUC), and Area Under Precision-Recall Curves (AUPR) were evaluated. Bold indicates the best performance

Model	Task^**a**^	Feature	10-fold CV RMSE					Acc.	Sen.	Spe.	AUC	AUPR
			Task1	Task2	Task3	Task4	Task5					
LASSO	ST	no group	0.8601	0.9221	1.0101	0.7578	0.6166	85.44%	79.03%	**93.55%**	0.8623	0.8648
SGL	ST	grouped	0.7931	0.8399	0.9157	0.6406	0.5328	85.93%	82.24%	90.62%	0.8647	0.8699
*ℓ*_1, 2_*MTL*	MT	no group	1.355	0.7982	0.7832	1.2654	0.6348	86.94%	83.67%	91.09%	0.8729	0.8788
*ℓ*_1, ∞_*MTL*	MT	no group	1.1364	0.7861	0.7748	0.9157	0.595	86.88%	83.98%	90.54%	0.8728	0.8792
MTL-SGL	MT	grouped	**0.7598**	**0.7106**	**0.714**	**0.5631**	**0.4863**	**87.28%**	**85.35%**	89.71%	**0.8772**	**0.8851**

^a^*ST* single task, *MT* multi-task

### Antigenic distance and map construction

Both HI-based and sequences-based antigen maps were constructed using AntigenMap (http://sysbio.cvm.msstate.edu/AntigenMap) [48]. AntigenMap was also used to generate an antigenic distance matrix from serologic data (HI data), as described elsewhere [48]. Specifically, a nuclear norm regularization–based method [48] was used to recover a low-rank data matrix for the HI table. The optimal parameter k for nuclear norm regularization was set to 1. The low-reactor threshold for low-rank matrix completion was set to 10, and a spectral clustering method was applied to identify antigenic clusters in antigenic maps as described elsewhere. In the antigenic maps, a threshold of 2 units of antigenic distance, representing a 4-fold HI titer change, was used as the threshold of antigenic variant detection [48].

### Phylogenetic analyses and molecular characterization

Phylogenetic analyses were performed using FastTree 2.1 [49] and RAxML v8 [50] and visualized by FigTree (http://tree.bio.ed.ac.uk/software/figtree/) and ggtree [51]; tree topologies were validated by Mr. Bayes3 [52]. The 3D structure of the HA protein of A/USSR/90/1977 virus was generated by SWISS-MODEL (https://swissmodel.expasy.org), and the protein structure was visualized by UCSF Chimera [53].

### Virus and virus preparation

A/Texas/36/1991 (H1N1), which was determined to be in the antigenic cluster A(H1N1)season1977-SG86, was propagated in MDCK cells. Viruses will be ultra-centrifuged as described elsewhere [54]. The HA of A/Texas/36/1991 (H1N1) was sequenced using sanger sequencing and used for glycopeptide mapping in the glycoproteomics analyses.

### Determination of the structure of the *N*-glycosylation of HA glycoproteins of each immunogen using glycoproteomics approaches

The viral samples purified through ultracentrifuge were subjected to proteomics, glycomics, and glycoproteomics analyses as described elsewhere [55]. Briefly, the samples were digested using trypsin, and proteolytic peptides and glycopeptides were then split into aliquots for performing proteomics and glycoproteomics. Proteomics samples were deglycosylated using PNGase *F*: released glycans were isolated for glycomics analyses; the deglycosylated peptides were analyzed to determine the site-occupancy. Glycoproteomics samples were incubated in the deglycosylation conditions without PNGase *F* (as a control for spontaneous deamidation at non-glycosylated asparagine residues), and the glycosylated peptides were analyzed for glycoproteomics to characterize the site-specific glycosylation patterns. All samples were subjected to LC-MS/MS analysis. The occupancy of glycosylation and site-specific glycosylation patterns were determined using GlycReSoft [56, 57].

## Results

### MTL-SGL model for quantifying antigenic distance using genomic sequences

Our long-term goal is to develop a genomic sequence–based method to quantify antigenic distances between influenza viruses and to understand the key residues driving antigenic evolution of influenza viruses. In this study, an MTL-SGL model was developed and then applied to the H1N1 IAVs. The model was used to identify genetic determinants from two types of features (i.e. sequence and *N*-glycosylation) affecting influenza virus antigenicity by learning the weights for each feature. A larger weight indicates a higher impact of this feature on influenza virus antigenicity. The unique *N*-glycosylation sites for HA of H1N1 IAVs are shown in Table S2. To avoid potential biases due to data integration processes, a MTL framework was proposed to handle multiple datasets by considering those from individual experiments as individual tasks. The MTL-SGL model comprised three integrated steps: data processing, multi-task feature learning, and antigenic distance prediction and antigenic map construction (Fig. 1).

The MTL-SGL model was trained on five individual tasks derived from three individual datasets (Table S1). During learning, we optimized three hyper parameters, λ_1_, λ_2_and λ_3_, in the MTL-SGL model by minimizing RMSE for each task through 10-fold cross-validation. Results showed that of the five models tested, our MTL-SGL model achieved the best performance with the combination of λ_1_ = 0.1, λ_2_ = 0.1, and λ_3_ = 5: an average RMSE of 0.6477 units, an average accuracy of 87.28% for identifying antigenic variants, and a sensitivity of 85.35% (Table 1; Figure S1).

We further compared the MTL-SGL method with two conventional multi-task models ,*ℓ*_1, 2_ MTL and *ℓ*_1, ∞_ MTL, and two conventional single-task models, LASSO and SGL [58–62]. Results showed that across all five testing models, MTL-SGL achieved the lowest RMSE across all five tasks (Table 1, Figure S2). Furthermore, MTL-SGL also achieved the highest accuracy and sensitivity (Table 1, Figure S2). These results demonstrate that the MTL-SGL model is effective for quantifying antigenicity and identifying antigenic variants for H1N1 IAVs.

### Antigenicity-associated residues and glycosylation sites derived by using the MTL-SGL model

By applying the MTL-SGL method to 2030 pairs of HI data for A(H1N1)season1977 viruses, A(H1N1)pdm09 viruses, and A(H1N1) swine influenza viruses (SIVs), we determined that among a total of 167 residues with variations, 78 features were associated with antigenicity of the viruses (Figure S3; Table S3 and S4). The 167 residues with variations consisted of 162 amino acid mutations and 5 *N*-glycosylation sites (Table S2). The 78 features consisted of 73 residues and 5 *N*-glycosylation sites. Among those 73 selected mutations, 46 are located in antibody binding sites (8 in Sa, 15 in Sb, 7 in Ca1, 7 in Ca2, and 8 in Cb), six (i.e., 130, 132, 183, 187, 191, and 222) were located in receptor binding sites, and 16 were located in regions outside the antibody and receptor binding sites (Figure S3, Table S3). Among these mutations, those at residues 71, 130, 141, 186, and 272 were among those with the highest weights.

Among the five non-conserved potential *N*-glycosylation sites, all of them were determined to affect antigenicity of H1N1 IAVs (Table S4). Sites 54, 125, and 160 were predicted with high weights, indicating changes on those sites could greatly change the antigenicity and have the potential to result in antigenic drift.

### Validation of *N*-glycosylation sites 54 and 125 in RU77 to SG86

During antigenic drift event from RU77 to SG86, the H1N1 virus gained two potential *N*-glycosylation sites at 54 and 125, both of which were predicted to play an important role in determining antigenic variations between RU77 and SG86. To determine whether *N*-glycosylation sites 54 and 125 are expressed *N*-linked glycans, we performed glycoproteomics analyses for HA proteins of A/Texas/36/1991(H1N1), which was clustered into SG86. Sequence analyses showed that 11 potential *N*-glycosylation sites, including 10 (NNST), 11(NSTD), 23(NVTV), 54(NCSV), 87(NGTC), 125(NHTV), 160(NLSK), 269(NASM), 287 (NSSL), 481(NGTY), and 540(NGSL), of which 54(NCSV) and 125(NHTV) are unique to the viruses in SG86 from RU77. In the mass spectrometry-based glycoproteomics analyses, eight of these 11 *N*-glycosylation sites except 10 (NNST), 11(NSTD), and 540(NGSL) were identified in the glycopeptides, and four sites were identified with *N*-linked glycan occupancy. Site 54(NCSV) has three types of complex *N*-glycans: HexNAc:2 Hex:5, HexNAc:2 Hex:6, HexNAc:2 Hex:7; site 125(NHTV) has HexNAc:5, Hex:5, Fuc:1; site 160 (NLSK) has HexNAc:2 Hex:7 or HexNAc:2; Hex:8; and site 269 (NASM) has HexNAc:2 Hex:7 or HexNAc:3 Hex:6 (Table 2). Of note, the *N*-glycosylation sites 54 (NCSV) and 125 (NHTV) were predicted to drive antigenic drift from RU77 to SG86; and 54 (NCSV), 125 (NHTV), 160 (NLSK) were predicted cause antigenic variations among swine IAVs. In summary, all three predicted *N*-glycosylating sites (i.e., 54, 125, and 160) affecting antigenic variations were validated to be with *N*-linked glycan complex occupancy.

**Table 2:**
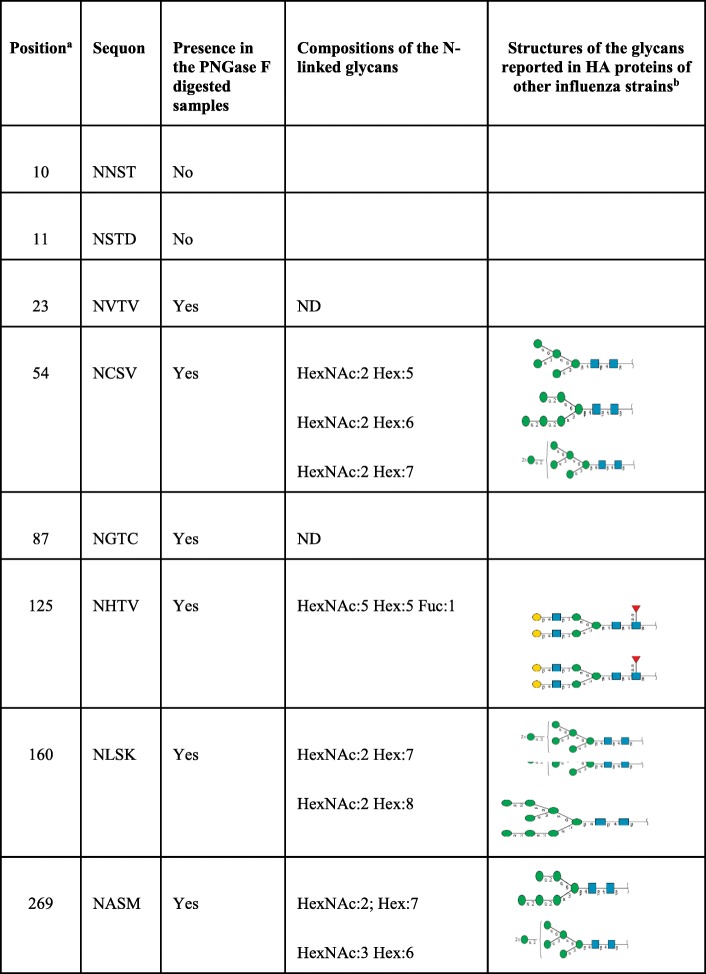
The occupancy analyses for *N*-linked glycans on the HA protein of A/Texas/36/1991(H1N1) using mass spectrometry

^a^The signal peptide was removed; ^b^The structures were obtained by searching the N-linked compositions against GlyConnect database (http: glyconnect.expasy.org)

### Large-scale profiling of antigenic evolution of H1N1 IAVs using only HA sequences

**Fig. 3:**
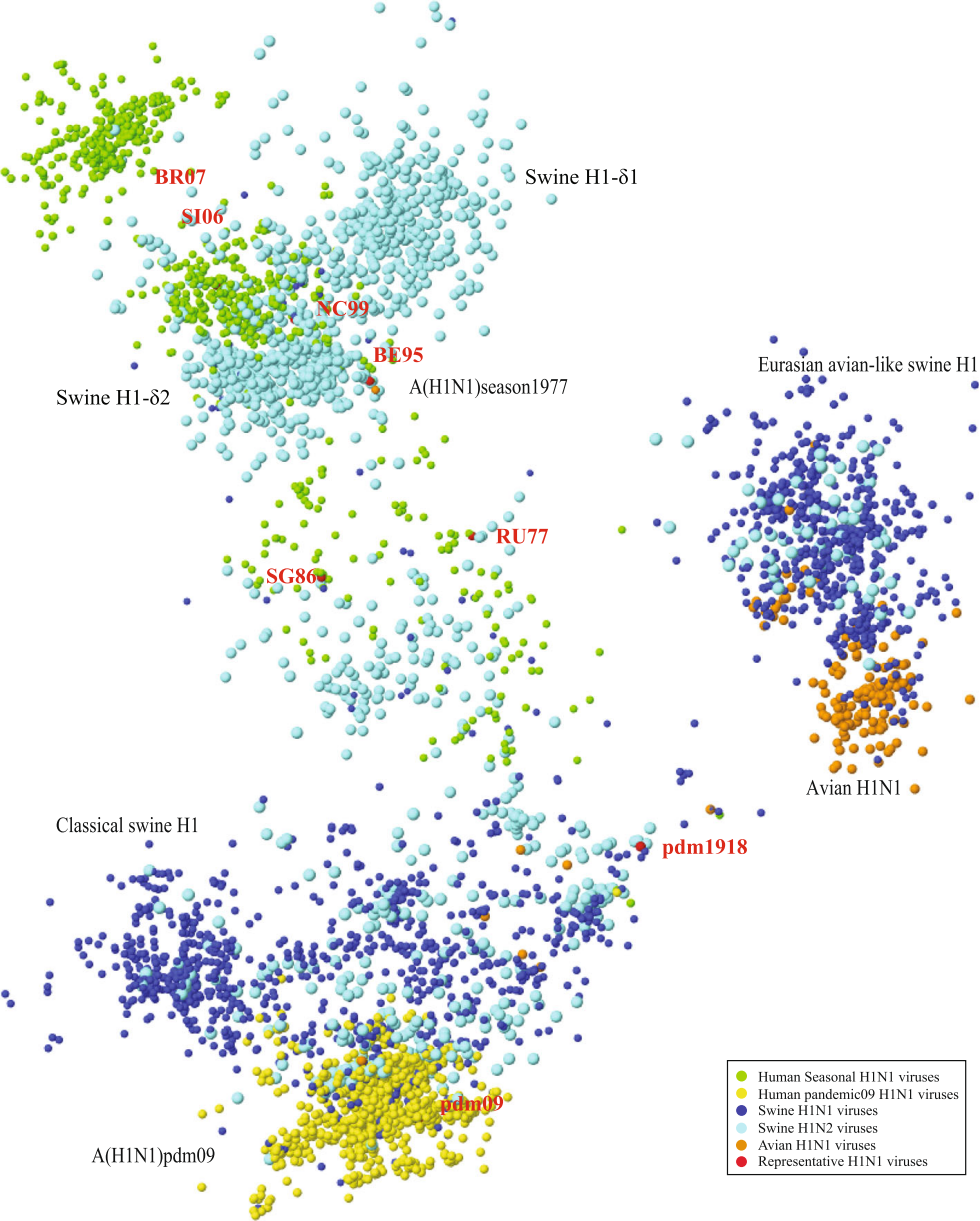
Large-scale sequence-based maps of 13,591 non-identical human, swine, and avian H1 influenza A viruses (IAVs). Pairwise antigenic distances were inferred from hemagglutinin subunit HA1 sequences by using a linear scoring function combined with weights generated from the MTL-SGL model. The map was generated from pairwise distances by using a multidimensional scaling algorithm. Antigenic cluster: BE95, A/Beijing/262/1995(H1N1)-like virus; BR07, A/Beijing/262/1995(H1N1)-like virus; NC99, A/New Caledonia/20/1999(H1N1)-like virus; pdm09, A(H1N1)pdm09-like virus; pdm1918, 1918 pandemic H1N1-like virus; RU77, A/USSR/90/1977(H1N1)-like virus; SG86, A/Singapore/6/1986(H1N1)-like virus; SI06, A/Solomon Islands/3/2006(H1N1)-like virus

By using the MTL-SGL model (Fig. 1), we quantified antigenic distances for a total of 13,591 non-identical HA sequences [1 A(H1N1)pdm1918, 1426 A(H1N1)season1977, 6483 A(H1N1)pdm09, 3052 swine H1N1, 1771 swine H1N2, and 858 avian H1N1 viruses], as described above, and then constructed a sequence-based H1N1 IAV–specific antigenic map (Fig. 3). A total of 14 antigenic clusters were identified (see details in Supplementary Information); they include 7 clusters in human H1 IAVs [i.e., A(H1N1)pdm1918 (1918 pandemic H1N1-like virus), seasonal H1N1 from 1930 to 1957 and 1977–2009, 4 antigenic clusters in A(H1N1)season1977 [i.e., we names RU77 (A/USSR/90/1977(H1N1)-like virus), SG86 (A/Singapore/6/1986(H1N1)-like virus), BE95/NC99 (A/Beijing/262/1995(H1N1)-like and A/New Caledonia/20/1999(H1N1)-like viruses), and SI06/BR07 (A/Singapore/6/1986(H1N1)-like virus and A/Solomon Islands/3/2006(H1N1)-like virus), and A(H1N1)pdm09 (A(H1N1)pdm09-like virus)]; 6 clusters in H1 SIVs (i.e., swine Eurasia avian-like H1N1, 3 clusters in classical swine H1 [α, β and γ], swine H1- *δ* 1, and swine H1- *δ* 2); and 1 cluster in avian H1N1 IAVs. Of note, the swine H1- *δ* 2 cluster and the human A(H1N1)season1977-BE95/NC99 cluster were located in the same antigenic cluster. In addition, IAVs in most of those antigenic clusters could associate with multiple hosts (e.g., spillovers between humans and swine).

### Sporadic spillovers of H1N1 IAVs among the interface of avian species, swine, and humans

**Fig. 4:**
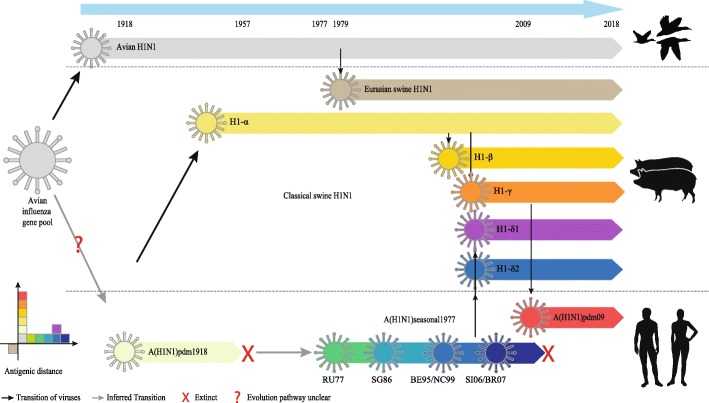
Large-scale antigenic evolution profile of H1 influenza A viruses in avian species, swine and humans. Different antigenic clusters are indicated by different colors. Colors of antigenic clusters indicate the antigenic profiles of corresponding clusters. As indicated in the key at the bottom left of the figure, a similar color indicates a small antigenic distance, and a dissimilar color indicates distinct antigenicity

Antigenic cartography clearly showed the spillovers among the avian, swine, and human interfaces. Antigenic data clearly demonstrated that avian H1N1 IAV was introduced into swine and has been enzootic in the Eurasian swine population since 1979, the first year a virus was isolated. Of interest, few antigenic variations were observed after H1N1 IAV was introduced into swine. Compared with a single spillover case of H1N1 IAV from swine to an avian species, a total of three spillover events were observed between the interface of swine and humans; one of these events was from swine to humans and two were from humans to swine (Fig. 4).

## Discussion

Protein *N*-glycosylation sites, known as sequons, bear a consensus sequence of Asn-X-Ser/Thr, where X is any amino acid except Pro [63]. Prior studies suggested that ~ 50% of N-X-S/T sequons are *N*-linked glycosylated [64, 65]. It has been well documented that changes in both mutations in protein sequences and glycosylation patterns affect antigenicity of influenza viruses [29]. However, to our knowledge, the major computational models used in those studies focused solely on changes in amino acids or treated glycosylation as the same type of feature as amino acid changes. Changes in glycosylation and changes in biophysical properties would logically be grouped into different biological processes and, thus, into different types of features. Therefore, a computational model considering different types of features is needed to better predict antigenicity using genomic sequences.

A few models and algorithms of sequence-based predictions of influenza antigenicity were proposed in the past few years. A few computational methods, such as the regression analyses [66, 67], decision tree [68], random forest regression and support vector regression [69], between variations in the serological data (e.g. HI titers) and mutations in HA protein sequences. Under the hypothesis that influenza antigenicity would be determined by a small number of features embedded in the influenza genomic sequence, especially the HA protein sequence and tertiary structure, a machine learning framework using sparse learning was developed and shown to be effective in determining residues associated with antigenicity of H3N2 and H5N1 IAVs [35–37]. To achieve a robust model for sequence-based prediction, various unique computational components have been developed to improve this framework, such as high-order models to investigate the effect of combinations of multiple individual mutations on antigenicity [37, 39] and multitask models [38] to avoid data integration required by conventional single task learning, which can lead artifical biases from the data integration steps. This MTL framework can be extremely important for large-scale modeling because more and more serologic data are generated by the rapid growth of experimental technology, and the robustness and effectiveness of MTL model handling multiple data sources (different reagents, protocols, labs) have been proved by a prior study on H3N2 influenza data [38]. Similar to the sparse learning model proposed by Cai et al. (2012) and Sun et al. (2013), Neher et al. (2016) adapted sparse learning model to identify residues associated with influenza viruses by correlating serological data and protein sequence data. In addition to protein features, the genetic distances and tree topologies in the phylogenetic trees were used as a penalty function in the model [70]. Harvey et al. developed a sparse Bayesian variable selection methods to study mutations in HA sequences affecting antigenic changes of H1N1 seasonal influenza (1997 through 2009) [28]. Nevertheless, none of these methods have considered multiple type of features (e.g. glycosylation) in predicting antigenicity of influenza viruses.

In this study, we developed and validated an MTL-SGL method, allowing for the training and learning of multiple categories of features and the integration of datasets from multiple sources (e.g., those generated using different reagents, supplies, and protocols) in the sparse learning process. Specifically, two types of features (protein sequence and *N*-glycosylation) were used; a multi-task framework was applied to strategically distribute multiple datasets as different tasks. Mathematically, MTL-SGL integrates Lasso (L1 norm), group lasso (L2 norm), and multi-task framework. The penalty combines with L1 and L2 norm regulate sparse at both the group and individual feature. Furthermore, multi-task penalty controls sparse across multiple tasks. Results from this study suggest the MTL-SGL model performed better than two conventional multi-task models (*ℓ*_1, 2_ MTL and *ℓ*_1, ∞_ MTL) and two conventional single-task learning models (SGL and LASSO) (Table 1). These results suggested that considering features from different biological properties as different groups improves model performance. This MTL-SGL method has potential scalability in feature categories and can be expanded to add other types of features in HA into the learning process, even those across other genomic segments (e.g., NA).

Although it is still developed on the same sparse learning framework we have developed in the past few years, MTL-SGL is unique and novel from any of those models reported in the literature. For example, the multitask model focused on learning task relationships along the task dimension but did not consider any structured sparsity over the feature dimension. In contrast, the MTL-SGL method in this manuscript considers the problem of learning group sparsity over features while allowing such underlying feature patterns to be shared across multiple tasks. Mathematically, by introducing the group sparsity over features in the MTL-SGL method, the formulated model involves one more non-smooth term in addition to the existing task sharing L1,2 / L1, ∞ penalty. This makes the optimization much more challenging in MTL-SGL than the multitask model, because we now have to manage both rows and columns of the entire parameter (which is a matrix) simultaneously instead of naively separating them. Instead, the method proposed in the multitask model did not suffer from the same problem, because it only focused on one dimension (the task). Hence, we have to devise a new smoothing proximal operator in the optimization algorithm to handle both the non-smooth task sharing term and the non-smooth group sparsity term for features, guaranteeing the convergence of the algorithm at the same time.

Through MTL-SGL based machine learning, a total of 73 residues have been identified as being associated with antigenicity in H1N1 IAVs. Among these residues, 9 (i.e. 153, 155, 163, 186, 187, 190, 194, 222, and 261) were reported under positive selection [71, 72]. In addition, a few residues (e.g., 43, 71, 130, 141, and 187) were reported to affect antigenicity of H1N1 IAVs by prior studies [28, 69]. Prior studies have demonstrated that changes of one or a small number of residues at antibody binding sites can lead to antigenic drift of H3N2 [23–26], and many of these mutations are located in or close to the HA receptor binding sites [73]. Of interest, similar to those in H3N2 IAVs [73], nine of H1N1 IAV antigenicity associated residues identified by MTL-SGL are located in (i.e., 130, 132, 183, 187, 191, and 222) or close (i.e. 186, 128, 127) to the HA receptor binding sites. On the other hand, MTL-SGL did identify 16 residues outside reported antibody binding site and receptor binding sites of HA to be associated with antigenic changes in H1N1 IAVs (Figure S3, Table S3).

In addition to those high-impact mutations, a few *N*-glycosylation sites were also considered to be highly associated with H1N1 IAV antigenic drift events. Results from the MTL-SGL model indicated that change of *N*-glycosylation sites 54 and 125 was the primary cause of the antigenic drift event RU77 → SG86, and both sites were validated to be occupied with *N*-linked glycans using mass spectrometry analyses (Table 2). Glycosylation has been well studied in regard to its effect on the antigenicity of various IAV subtypes, such as H1N1 viruses [29, 74], H5N1 viruses [75], and H3N2 viruses [76]. For H1N1 viruses, a total of 20 potential *N*-glycosylation patterns have been observed in HA subunit (HA1) protein sequences [7]. *N*-glycosylation sites, especially 54 and 125, were confirmed to play an important role in antigenic diversity by masking antigenic sites [29]. In our study, mutations at three non-conserved *N*-glycosylation sites (54, 125, and 160) were identified to greatly affect antigenic changes in H1N1 IAVs. However, not all the glycosylation sites contribute equally to antigenicity. Our results show that changing *N*-glycosylation site 125 has a much bigger effect on antigenicity than a change in any other site. Because prior studies demonstrate that HA glycosylation could affect antigenicity and pathogenicity of human H1N1 IAVs by masking antigenic sites and eliciting protective immune response, HA glycosylation, including the structure and composition of the *N*-glycans present at each site, is extremely important for sequence-based antigenicity quantification and vaccine strain selection; thus, it should always be considered as an essential factor in sequence-based predicting models. Current model only considers the presence or absence of the *N*-glycans at the HA but not the structure and composition of the *N*-glycans present at each site. As future study, we hope to integrate the glycoproteomics approaches to define patterns of the structure and composition of the *N*-glycans for each position, and these features will be integrated and used to optimize the computational model.

On the other hand, it is not a trivial task to predict the *N*-linked glycosylation site occupancy from the protein sequences inferred from protein sequences. In past decades, a number of computational methods have been developed for predicting *N*-linked glycosylation sites given a protein sequence. Most of these methods were based on machine learning approaches, such as neural network [77], support vector machine [78], and random forest [47, 79, 80]. These methods consider neighboring sequences and protein structure [47, 80] and assign a probability for a *N*-linked glycosylation site for each position. To be the best of our knowledge, little is known the specific mutation patterns (in addition to sequon) are associated with acquisition or loss of a *N*-glycosylation site in influenza viruses.

By using the MTL-SGL model, we constructed a large-scale antigenic evolution profile for a total of 13,591 H1N1 IAVs to represent the antigenic evolution history of H1N1 IAVs in the past 100 years (Fig. 4). Results showed that all H1N1 IAVs in swine and humans seem to be antigenically associated with A(H1N1)pdm1918, which was proposed to have originated directly from an avian species [81]. The antigenic evolution profile suggested that avian H1N1 IAVs showed less antigenic diversity than swine and human H1N1 IAVs. However, after being introduced into swine and human populations, IAVs had different degrees of antigenic drift: the antigenic changes in humans (e.g., average antigenic distances between RU77 cluster in 1977 and SI06/BR07 cluster in 2007 was 8.7513 [±0.9110]) were much more extensive than those in swine population (e.g., average antigenic distances between SIV H1- *α* cluster and H1- *γ* cluster = 4.5198 [± 0. 8875]). Results also showed that multiple antigenic clusters (i.e., H1- *α*, H1- *β*, and H1- *γ*) were maintained in swine for years but usually only one antigenic cluster could be maintained in humans. Of note, A(H1N1)season1977 IAVs co-circulated with A(H1N1)pdm09 IAVs for 1 year after A(H1N1)pdm09 emerged and then were rapidly eradicated in 2010. The sporadic spillovers of H1N1 IAVs at the avian–swine interface can lead to enzootic antigenic variants in swine; the sporadic spillovers of H1N1 at the human–swine interface could potentially introduce an enzootic antigenic variant among swine or a pandemic virus among humans. Although spillovers among avian, swine, and human interfaces are sporadic, antigenic variants present in avian species and swine continue to present risks to human public health.

## Conclusions

In summary, in this study, we developed and validated a sequence-based influenza antigenic variant detection method for H1 IAVs. This method is complementary to conventional serologic assays, such as HI and NI assays, which usually are medium or low throughput. Because genomic sequences can be achieved directly from clinical samples without virus isolation, this method can be high throughput and applied in large-scale antigenic characterization in influenza vaccine strain selection.

## Supplementary information

**Additional file 1.**

## Data Availability

Sequences and serologic data can be accessed at https://github.com/InfluenzaSystemsBiology/MTL-SGL.

## Abbreviations

ADMM: Alternating direction method of multipliers
AUC: Area Under Receiver Operating Characteristic Curves
AUPR: Area Under Precision-Recall Curves
Acc: Average accuracy
Sen: Average sensitivity
Spe: Average specificity
HA: Hemagglutinin
MTL: Multi-task learning
(LASSO), MTL-SGL: Multi-task learning sparse group least absolute shrinkage and selection operator
IAV: Influenza A viruses
NA: Neuraminidase
RMSE: Rooted mean square error
