# Supplementary material for: Multi-task learning sparse group lasso: a method for quantifying antigenicity of influenza A(H1N1) virus using mutations and variations in glycosylation of Hemagglutinin
Source: BMC Bioinformatics. 2020 May 11;21:182. doi: 10.1186/s12859-020-3527-5 (PMC7216668; doi:10.1186/s12859-020-3527-5)
Supplement: Supplementary file 1 — Additional file 1. [file 12859_2020_3527_MOESM1_ESM.pdf]

Supplementary Information

Title: Multi-Task Learning Sparse Group Lasso: a Method for Quantifying Antigenicity of Influenza A(H1N1) Virus using Mutations and Variations in Glycosylation of Hemagglutinin

Lei Li[1], Deborah Chang[2], Lei Han[1,3], Xiaojian Zhang[1,4,5,6], Joseph Zaia[2], and Xiu-Feng Wan[1,4,5,6,7,8,*]

[1]Department of Basic Sciences, College of Veterinary Medicine, Mississippi State University, Mississippi State, MS, USA; [2]Center for Biomedical Mass Spectrometry, Department of Biochemistry, Boston University School of Medicine, Boston, MA, USA; [3]Tencent AI Lab, Shenzhen, China; [4]Department of Molecular Microbiology and Immunology, School of Medicine, University of Missouri, Columbia, MO, USA; [5]Missouri University Center for Research on Influenza Systems Biology (CRISB), University of Missouri, Columbia, MO, USA; [6]Bond Life Sciences Center, University of Missouri, Columbia, MO, USA; [7]Department of Electrical Engineering & Computer Science, College of Engineering, University of Missouri, Columbia, MO, USA; [8]MU Institute for Data Science and Informatics, University of Missouri, Columbia, MO, USA;

*To whom correspondence should be addressed: Dr. Xiu-Feng Wan, wanx@missouri.edu.

Abstract: 232 words; **Main Text**: 5,494 words.

Number of Figures: 4; **Number of Tables**: 2; **Supplementary Information**: 1 file.

Supplementary Results

Antigenic analyses of A(H1N1) viruses from 1918 to 2018. The within-cluster antigenic distances among these 14 antigenic clusters vary to different extents: A(H1N1)pdm1918 (within antigenic distance of 1.6333 ± 0.8644), 2) seasonal H1N1 from 1930–1957 (1.4147 ± 1.0350), 3) A(H1N1)season1977-RU77 (2.4103 ± 2.0888), 4) A(H1N1)season1977-SG86 (1.4870 ± 0.7603), 5) A(H1N1)season1977-BE95/NC99 (2.3170 ± 1.6981), 6) A(H1N1)season1977-SI06/BR07 (2.0807 ± 0.9592), 7) A(H1N1)pdm09 IAVs (0.5696 ± 0.3932), 8) SIV-H1- $\boldsymbol{\alpha}$ (0.8878 ± 0.5501), 9) SIV-H1- $\boldsymbol{\beta}$ (2.6547 ± 1.5614), 10) SIV-H1- $\boldsymbol{\gamma}$ (1.2696 ± 0.9399), 11) SIV-H1-$\boldsymbol{\delta}$1 (1.4408 ± 1.6637), 12) SIV-H1-$\boldsymbol{\delta}$12 (1.3685 ± 1.5802), 13) SIV Eurasia avian-like H1 (3.2044 ± 1.9459), and 14) AIV-H1 (1.4412 ± 1.7569).

Average antigenic distances between RU77-SG86, SG86-BE95/NC99, and BE95/NC99-SI06/BR07 are 4.8224 (±1.4157), 3.8771 (±1.3613), and 3.2259 (±1.6391), respectively (Figure S5G–I). Within those 4 antigenic clusters, the MTL-SGL model suggested that change of N-glycosylation sites 54 and 125 and mutation E186G drove the antigenic drift from RU77 to SG86; that deletion of K130 (130DEL) and mutations F71I, R43L, and I57V resulted in antigenic drift from SG86 to BE95/NC99; and that mutations K141E and D187N led to antigenic drift from BE95/NC99 to SI06/BR07.

For H1N1 SIVs, the average antigenic distances are 4.0853 (±0.9354), 4.2436 (±0.4907), and 4.5198(±0.8875) between H1-$\boldsymbol{\alpha}\ \boldsymbol{and}\ \boldsymbol{H1}-\boldsymbol{\beta}$, H1-$\boldsymbol{\beta}\ \boldsymbol{and}\ \boldsymbol{H1}-\boldsymbol{\gamma},$ and H1-$\boldsymbol{\alpha}\ \boldsymbol{and}\ \boldsymbol{H1}-\boldsymbol{\gamma}$, respectively. Results from the MTL-SGL model suggested that mutations V73A, R130K, and E127D drove the antigenic drift from swine H1-$\boldsymbol{\alpha}$ to H1-$\boldsymbol{\beta}$; mutation P271S, A141T, and R130K drove the antigenic drift from swine H1-$\boldsymbol{\beta}$ to H1-$\boldsymbol{\gamma}$; and mutations P271S, 130DEL, and A141T resulted in antigenic drift between H1-$\boldsymbol{\alpha}$ and H1-$\boldsymbol{\gamma}$. In addition, changes of N-glycosylation sites 54 (NCSV), 125 (NHTV), and 160 (NLSK) and mutations K43L and V272M were responsible

for the antigenic difference between $\boldsymbol{H1-\delta 1}$ and three other genetic clusters, H1-$\boldsymbol{\alpha}$, $\boldsymbol{H1-\beta}$, $\boldsymbol{and\ H1-\gamma}$. Cluster $\boldsymbol{H1-\delta}$ was predicted to be distinct from those clusters; the average antigenic distances are 11.9143 (±0.2502) between clusters $\boldsymbol{\delta}$ and $\boldsymbol{\alpha}$, 12.1317 (±0.2221) between clusters $\boldsymbol{\delta}$ and $\boldsymbol{\beta}$, and 12.4823 (±0.4326) between clusters $\boldsymbol{\delta}$ and $\boldsymbol{\gamma}$, respectively (Figure 4A).

Antigenic cartography suggested that avian H1N1 and Eurasia avian-like H1N1 IAVs are antigenically close to each other; the average antigenic distance between two clusters is 4.6653 (±1.2592) (Figure S5B). The avian H1N1 IAVs from North America and those from Europe are antigenically close to each other in the antigenic map; the average antigenic distance between those two genetic clades is 1.0644 (±0.6854), which indicates that there is little antigenic variation in avian H1N1 IAVs (Figure S5B).

The H1N1 viruses from avian species, swine, and humans have been well documented to be genetically associated with A(H1N1)pdm1918 and clustered into multiple major genetic lineages, including 1) avian H1N1 and swine Eurasia H1N1 belong to the same lineage, 2) human A(H1N1)season1977, swine H1- $\boldsymbol{\delta}$1, and swine H1- $\boldsymbol{\delta}$2 belong to the same lineage; and 3) all other H1N1 viruses, including human A(H1N1)pdm09 and the $\boldsymbol{\alpha, \beta, \gamma}$ cluster of swine classical H1N1, belong to the same lineage (Figure S4) (1-3). In the past decades, antigenic characterization has been individually performed for swine H1N1 IAVs (3, 4) and human A(H1N1)season1977 IAVs (5). However, a large-scale antigenic characterization is difficult due to the detection limits (distinct viruses usually do not react to each other in HI assays) and the labor/resource intensity of serologic assays. By using the MTL-SGL model, we constructed a large-scale antigenic evolution profile for a total of 13,591 H1N1 IAVs to represent the antigenic evolution history of H1N1 IAVs in the past 100 years (Figure 4). Results showed that all H1N1 IAVs in swine and humans seem to be antigenically associated with A(H1N1)pdm1918, which was proposed to have originated directly from an avian species (6). Of note, A(H1N1)pdm1918 and avian H1N1 IAVs are antigenically dissimilar (Figure 3), and the antigenic evolution of

A(H1N1)pdm1918 prior to 1918 is still unclear. A(H1N1)pdm1918 is antigenically closer to A(H1N1)pdm09 IAVs than to A(H1N1)season1977. The average antigenic distance (predicted) is 4.2094 (±0.2889) between A(H1N1)pdm1918 and A(H1N1)pdm09 (Figure S5D). A(H1N1) season1977 viruses are antigenically distinct from A(H1N1)pdm1918 and A(H1N1)pdm09 viruses; average predicted antigenic distances are 11.3261 (±0.5875) between A(H1N1)season1977 and A(H1N1)pdm09 viruses and 9.9311 (±0.6627) between A(H1N1) season1977 and A(H1N1)pdm1918 viruses (Figure S5E, F).

Validation of antigenic distances among H1N1 IAVs. To confirm those predicted antigenic distances, we adopted HI data from a prior study (7) that included representative viruses for A(H1N1)pdm09, A(H1N1)season1977, and A(H1N1). The HI-based antigenic distance between A(H1N1)pdm1918 and A(H1N1)pdm09 is 3 units (HI titer drop from 2,560 to 320); the distance between A(H1N1)season1977 and A(H1N1)pdm09 is > 5.75 units (drop from 640/320 to < 10); and the distance between A(H1N1) season1977 and A(H1N1)pdm1918 is > 5.75 units (drop from 640/320 to < 10).

Supplementary Tables

Table S1 Detailed information for all training datasets in this study.

**Dataset**	**Time**	**Genetic Lineage**	**Viruses**	**Sera**	**HI Titer**
1	1977-2009	Human A(H1N1)season1977	115	53	1062
2	1998-2009	Swine H1N1, A(H1N1)season1977, A(H1N1)pdm09	14	8	104
3	1930-2008	Swine H1N1	24	36	864

Table S2. Non-conserved N-glycosylation sites and amino acid residues used for learning in this study.

**Feature**	**Sites/Residues**
N-glycosylation sites	54, 125, 127, 155, 160
Amino Acid Residues	2,3,17,19,21,23,24,32,35,36,38,40,43,45,47,51,54,56,57,61,66,68,69,71,72,73,74,80,82,83,84,85,86,89,94,96,97,104,109,112,113,119,120,121,125,126,127,128,129,130,132,133,134,135,137,138,139,141,142,146,149,152,153,155,156,157,160,161,162,163,166,168,170,175,176,178,179,183,184,185,186,187,189,190,191,193,194,195,196,197,199,200,202,203,205,207,208,209,211,215,216,222,224,227,228,232,233,234,239,241,245,249,250,252,253,256,257,258,259,260,261,262,267,269,270,271,272,273,274,276,277,278,281,282,283,284,285,286,287,288,289,290,291,292,293,294,295,296,297,298,299,300,301,302,308,310,311,313,314,315,321,324

Table S3. Global weights for features selected by MTL-SGL model (sorted by absolute values of weights). Residues reported under positive selection (8, 9) were indicated by *.

Feature	Global	ABS&RBS	Type
272	0.909031417		Mutation
160(N)	0.736203263	Sb	N-linked Glycosylation Site
290	0.615497282		Mutation
269	-0.6071		Mutation
125(N)	0.517732131	Sb	N-linked Glycosylation Site
57	0.484361027		Mutation
141	0.470879862	Sa	Mutation
222*	0.463863995	RBS	Mutation
132	0.461702436	RBS	Mutation
112	0.445387063		Mutation
43	0.444866801	Ca1	Mutation
80	0.419401769	Cb	Mutation
187*	0.410432598	Sb, RBS	Mutation
54(N)	0.409534591	Cb	N-linked Glycosylation Site
35	0.409121005	Ca1	Mutation
130	0.398599047	RBS	Mutation
215	0.375419306	Ca2	Mutation
71	0.36329834		Mutation
121	0.325524941		Mutation
315	0.323474095		Mutation
216	0.322062218	Ca2	Mutation
157	0.303850901	Sb	Mutation
271	0.297818095	Ca1	Mutation
127	0.29199604	Sa	Mutation
2	0.287465112		Mutation
189	0.280358971	Sb	Mutation
250	0.269296789		Mutation
128	0.267554762	Sa	Mutation
205	0.262560191	Ca2	Mutation
186*	0.25661292	Sb	Mutation
94	0.256106084		Mutation

85	0.255499207		Mutation
74	0.238338235	Cb	Mutation
301	0.233850378		Mutation
40	0.232150024	Ca1	Mutation
184	0.230308637	Sb	Mutation
194*	0.222508519	Sb	Mutation
277	0.219692754	Ca1	Mutation
253	0.203670647		Mutation
142	0.197177913	Sa	Mutation
153*	0.183301641		Mutation
183	0.180442501	Sb, RBS	Mutation
47	0.180427869		Mutation
89	0.173001952	Ca2	Mutation
310	0.171316151	Ca1	Mutation
211	0.170556808	Ca2	Mutation
166	0.164752547		Mutation
129	0.164051612	Sa	Mutation
267	0.163782694		Mutation
127(N)	0.163737806	Sa	N-linked Glycosylation Site
191	0.16192203	Sb, RBS	Mutation
283	0.148029684		Mutation
84	-0.147713546	Cb	Mutation
175	0.144607508		Mutation
261*	0.142210121		Mutation
96	0.138245345	Ca2	Mutation
190*	0.130657732	Sb	Mutation
258	0.126715863	Cb	Mutation
208	0.124251922		Mutation
161	0.122251507	Sb	Mutation
36	0.118806971	Ca1	Mutation
72	0.117943588	Cb	Mutation
82	0.113957015		Mutation
134	0.102046752		Mutation
252	0.10058056		Mutation
149	-0.098882505	Sa	Mutation

66	0.096433055	Cb	Mutation
155(N)*	0.094039003	Sb	N-linked Glycosylation Site
260	0.083834933	Cb	Mutation
135	0.076764447	Sa	Mutation
168	0.07495717	Ca2	Mutation
146	0.06297015		Mutation
156	0.06279503	Sb	Mutation
162	0.061401739	Sb	Mutation
273	0.056131632	Ca1	Mutation
232	-0.040207953		Mutation
120	0.020498478		Mutation
163*	0.017206527		Mutation

Table S4. Local weights for features of each task selected by MTL-SGL model (sorted by absolute values of weights). Residues reported under positive selection (8, 9) were indicated by *.

Task1		Task2		Task3		Task4		Task5	
**Feature**	**Weights**	**Feature**	**Weights**	**Feature**	**Weights**	**Feature**	**Weights**	**Feature**	**Weights**
125(N)	0.9250	35	0.6084	141	1.1607	125(N)	1.6860	89	0.3217
54(N)	0.7570	130	0.5196	57	0.6990	160(N)	1.6328	127	0.2920
186*	0.5295	71	0.5175	290	0.6155	272	0.9090	130	0.2794
222*	0.4639	43	0.5074	128	0.4745	71	0.3762	74	0.2725
216	0.3830	271	0.4920	132	0.4617	267	0.2724	186*	0.2487
277	0.3354	80	0.4841	94	0.4567	250	0.2693	35	0.2098
205	0.3339	141	0.4413	112	0.4454	258	0.1985	216	0.2096
121	0.3255	187*	0.3831	315	0.4406	141	0.1826	128	0.1914
127(N)	0.1637	215	0.3754	187*	0.4378	211	0.1706	205	0.1912
135	0.0768	253	0.3210	130	0.3968	283	0.1591	71	0.1651
		74	0.2978	71	0.3943	186*	0.1527	129	0.1641
		153*	0.2797	43	0.3823	271	0.1036	283	0.1370
		57	0.2697	216	0.3736	260	0.0838	267	0.1361
		85	0.2555	80	0.3547			120	0.1274
		160(N)	0.2444	160(N)	0.3315			142	0.1067
		183	0.2241	157	0.3039			141	0.0988
		191	0.2072	142	0.2876			96	0.0953
		315	0.2063	2	0.2875			156	0.0628
		66	0.1578	189	0.2804			273	0.0561
		186*	0.1430	175	0.2427			162	0.0438
		89	0.1376	301	0.2339				
		128	0.1367	40	0.2322				
		166	0.1271	184	0.2303				
		161	0.1223	194*	0.2225				
		84	0.1075	261*	0.2181				
		134	0.1020	190*	0.2148				

		155(N)*	0.0940	186	0.2092				
		163*	0.0884	166	0.2024				
		252	0.0709	96	0.1812				
		261*	0.0664	47	0.1804				
		72	0.0662	310	0.1713				
		146	0.0633	72	0.1697				
		94	0.0555	74	0.1448				
		175	0.0465	183	0.1368				
		190*	0.0465	252	0.1303				
		125(N)	-0.3739	208	0.1243				
				36	0.1188				
				191	0.1166				
				82	0.1140				
				277	0.1040				
				153*	0.0869				
				253	0.0864				
				267	0.0829				
				162	0.0790				
				168	0.0750				
				146	0.0626				
				54(N)	0.0621				
				89	0.0597				
				258	0.0549				
				66	0.0350				
				232	-0.0402				
				163*	-0.0540				
				120	-0.0864				
				149	-0.0989				
				125(N)	-0.1662				
				84	-0.4029				

		269 -0.6071		

Table S5. Antigenic variations of A(H1N1)pdm09 viruses from 2009 to 2016. High responder (High.) indicate < 4-fold lower than homologous titer to vaccine strain; Medium responder (Medium.) indicate = 4-fold lower than homologous titer to vaccine strain; Low responder (Low.) indicate ≥ 4-fold lower than homologous titer to vaccine strain. Number (No.) and percentage (Pct.) of viruses were listed for each time period.

Source	High.		Medium.		Low.		Total	Time period
	No.	Pct.	No.	Pct.	No.	Pct.		
2010/09/26	1763	96.13%	66	3.60%	5	0.27%	1834	Mar 2009 - Aug 2009
2011/02/14	2107	95.34%	92	4.16%	11	0.50%	2210	Sep 2009 - Feb 2010
2011/09/26	526	94.77%	22	3.96%	7	1.26%	555	Mar 2010 – Aug2010
2012/02/20	862	93.49%	52	5.64%	8	0.87%	922	Sep 2010 – Feb2011
2012/09/17	504	94.92%	19	3.58%	8	1.51%	531	Mar 2011 – Aug2011
2013/02/18	466	91.19%	34	6.65%	11	2.15%	511	Sep 2011 – Feb2012
2013/02/18	455	80.53%	76	13.45%	34	6.02%	565	Mar2012 – Aug2012
2013/09/23	343	84.28%	46	11.30%	18	4.42%	407	Aug2012 – Jan 2013
2014/08/12	641	95.25%	16	2.38%	16	2.38%	673	Feb 2013 – Aug2013
2015/02/23	1824	98.86%	16	0.87%	5	0.27%	1845	Sep 2013- Jan2014
2015/02/23	758	98.57%	8	1.04%	3	0.39%	769	Feb 2014 – Aug2014
2015/09/21	95	98.96%	1	1.04%	0	0.00%	96	Sep 2014 – Jan 2015
2016/02/22	244	93.13%	13	4.96%	5	1.91%	262	Feb2015 - Aug2015
2016/09/26	738	99.06%	6	0.81%	1	0.13%	745	Sep 2015 - Jan 2016
2016/09/26	1134	98.61%	12	1.04%	4	0.35%	1150	Feb 2016 - Sep 2016

Supplementary Figures

Figure S1. Optimization of parameters in the MTL-SGL machine learning models. A, B and C showed he model performance versus the parameter λ_1, λ_2 and λ_3 in multi-task feature learning, respectively.

Figure S2. Performance evaluation of the multi-task learning (MTL) methods (including the MTL-SGL, $\ell_{1,2}$ MTL and $\ell_{1,\infty}$ MTL), and the single task learning methods (including Lasso regression and sparse group lasso regression). The rooted mean square error (RMSE), Average accuracy (Acc.), Average sensitivity (Sen.), and Average specificity (Spe.) were evaluated.

Figure S3. The 3D structure of the HA protein with identified key mutations and *N*-glycosylation sites. As indicated in figure legends, different colors indicate different antibody binding sites (ABS) Sa, Sb, Ca1, Ca2, Cb and regions outside the ABS. Receptor binding sites (RBS) and *N*-glycosylation sites were also highlighted in different color.

Figure S4. Phylogenetic tree for all H1 viruses (including subtype H1N1 and H1N2) circulated in human, swine and avian. H1 IAVs from different host were highlighted in different colors as indicated in the legend. A representative virus of A(H1N1)pdm1918 was indicated on the tree. Genetic clades/lineages were indicated to the right of the tree.

Figure S5. Sequence-based maps for individual antigenic drifts of human, swine, and avian influenza A(H1N1) viruses. Different antigenic clusters are indicated by different colors; colors in each panel are independent of those in other panels. Red dots indicate representative viruses. A) $\alpha, \beta, \gamma,$ and δ clusters of classical swine H1N1 viruses. B) Avian H1N1 cluster and swine Eurasia H1N1 cluster. C) Swine classical H1N1 cluster and swine Eurasia H1N1 cluster. D) Human A(H1N1)pdm09 cluster and pandemic 1918 A(H1N1) [hereafter referred to as A(H1N1)pdm1918] cluster. E) Human seasonal 1977 A(H1N1) [hereafter referred to as A(H1N1)season1977] cluster and A(H1N1)pdm1918 cluster. F: Human A(H1N1)season1977 cluster and A(H1N1)pdm09 cluster. G: RU77 cluster and SG86 cluster in A(H1N1)season1977 viruses. H: SG86 cluster and BE95/NC99 cluster in A(H1N1)season1977 viruses. I: BE95/NC99 cluster and SI06/BR07 cluster in A(H1N1)season1977 viruses. Antigenic cluster: BE95, A/Beijing/262/1995(H1N1)-like virus; BR07, A/Beijing/262/1995(H1N1)-like virus; NC99, A/New Caledonia/20/1999(H1N1)-like virus; pdm09, A(H1N1)pdm09-like virus; pdm1918, 1918 pandemic H1N1-like virus; RU77, A/USSR/90/1977(H1N1)-like virus; SG86, A/Singapore/6/1986(H1N1)-like virus; SI06, A/Solomon Islands/3/2006(H1N1)-like virus.

References

1. Lewis NS, Russell CA, Langat P, Anderson TK, Berger K, Bielejec F, et al. The global antigenic diversity of swine influenza A viruses. Elife. 2016;5:e12217.
2. Morens DM, Taubenberger JK, Fauci AS. The persistent legacy of the 1918 influenza virus. New England Journal of Medicine. 2009;361(3):225-9.
3. Shu B, Garten R, Emery S, Balish A, Cooper L, Sessions W, et al. Genetic analysis and antigenic characterization of swine origin influenza viruses isolated from humans in the United States, 1990–2010. Virology. 2012;422(1):151-60.
4. Lorusso A, Vincent AL, Harland ML, Alt D, Bayles DO, Swenson SL, et al. Genetic and antigenic characterization of H1 influenza viruses from United States swine from 2008. Journal of General Virology. 2011;92(4):919-30.
5. Bedford T, Suchard MA, Lemey P, Dudas G, Gregory V, Hay AJ, et al. Integrating influenza antigenic dynamics with molecular evolution. Elife. 2014;3:e01914.
6. Taubenberger JK, Reid AH, Janczewski TA, Fanning TG. Integrating historical, clinical and molecular genetic data in order to explain the origin and virulence of the 1918 Spanish influenza virus. Philos Trans R Soc Lond B Biol Sci. 2001;356(1416):1829-39.
7. Manicassamy B, Medina RA, Hai R, Tsibane T, Stertz S, Nistal-Villán E, et al. Protection of mice against lethal challenge with 2009 H1N1 influenza A virus by 1918-like and classical swine H1N1 based vaccines. PLoS Pathog. 2010;6(1):e1000745.
8. Shen J, Ma J, Wang QJPo. Evolutionary trends of A (H1N1) influenza virus hemagglutinin since 1918. 2009;4(11):e7789.
9. Kirkpatrick E, Qiu X, Wilson PC, Bahl J, Krammer FJSr. The influenza virus hemagglutinin head evolves faster than the stalk domain. 2018;8(1):10432.

Figure S1

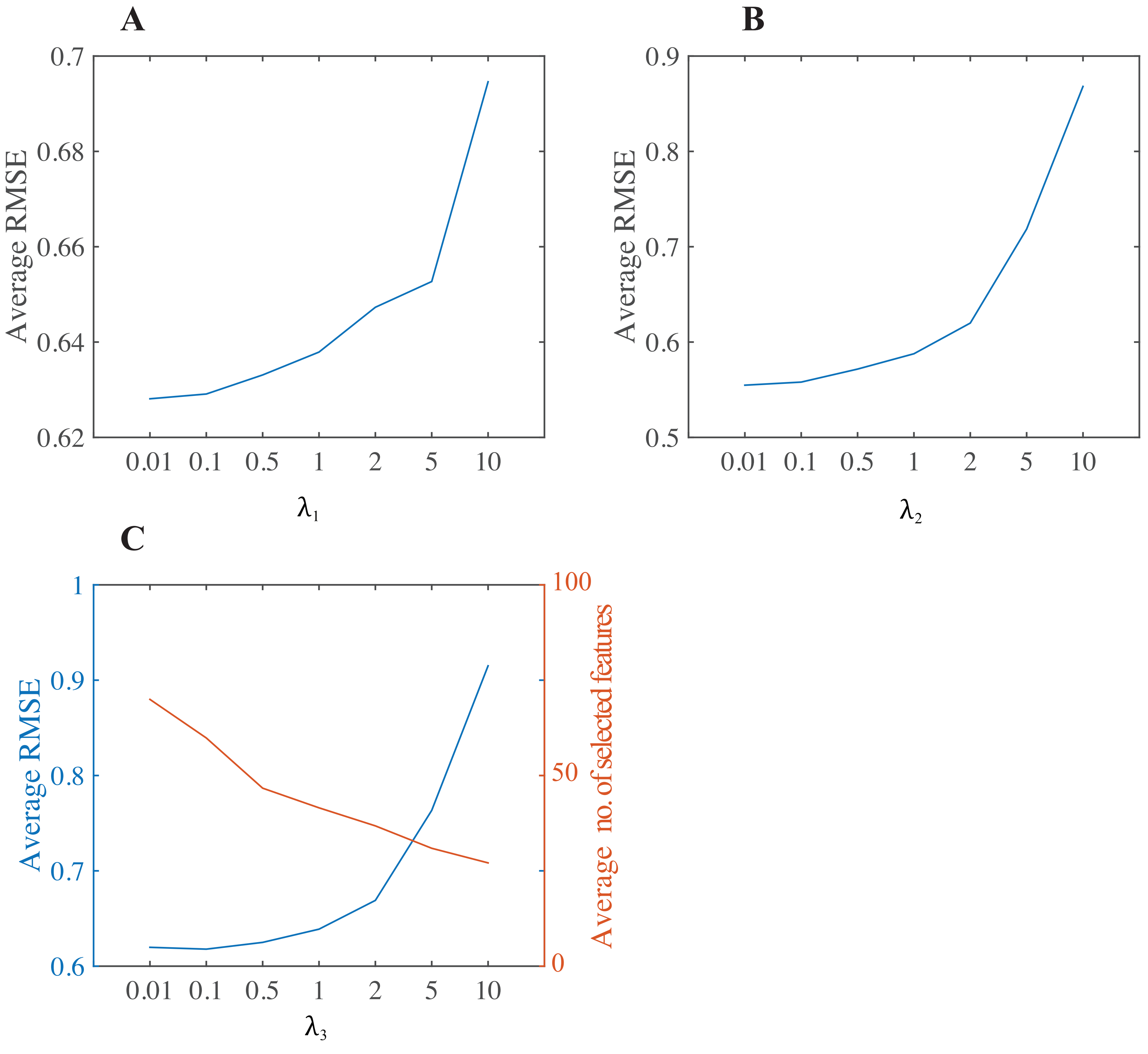
A
0.7
0.68
0.66
0.64
0.62
Average RMSE
0.01 0.1 0.5 1 2 5 10
λ1
B
0.9
0.8
0.7
0.6
0.5
Average RMSE
0.01 0.1 0.5 1 2 5 10
λ2
C
1
0.9
0.8
0.7
0.6
Average RMSE
100
50
0
Average no. of selected features
0.01 0.1 0.5 1 2 5 10
λ3

Figure S2

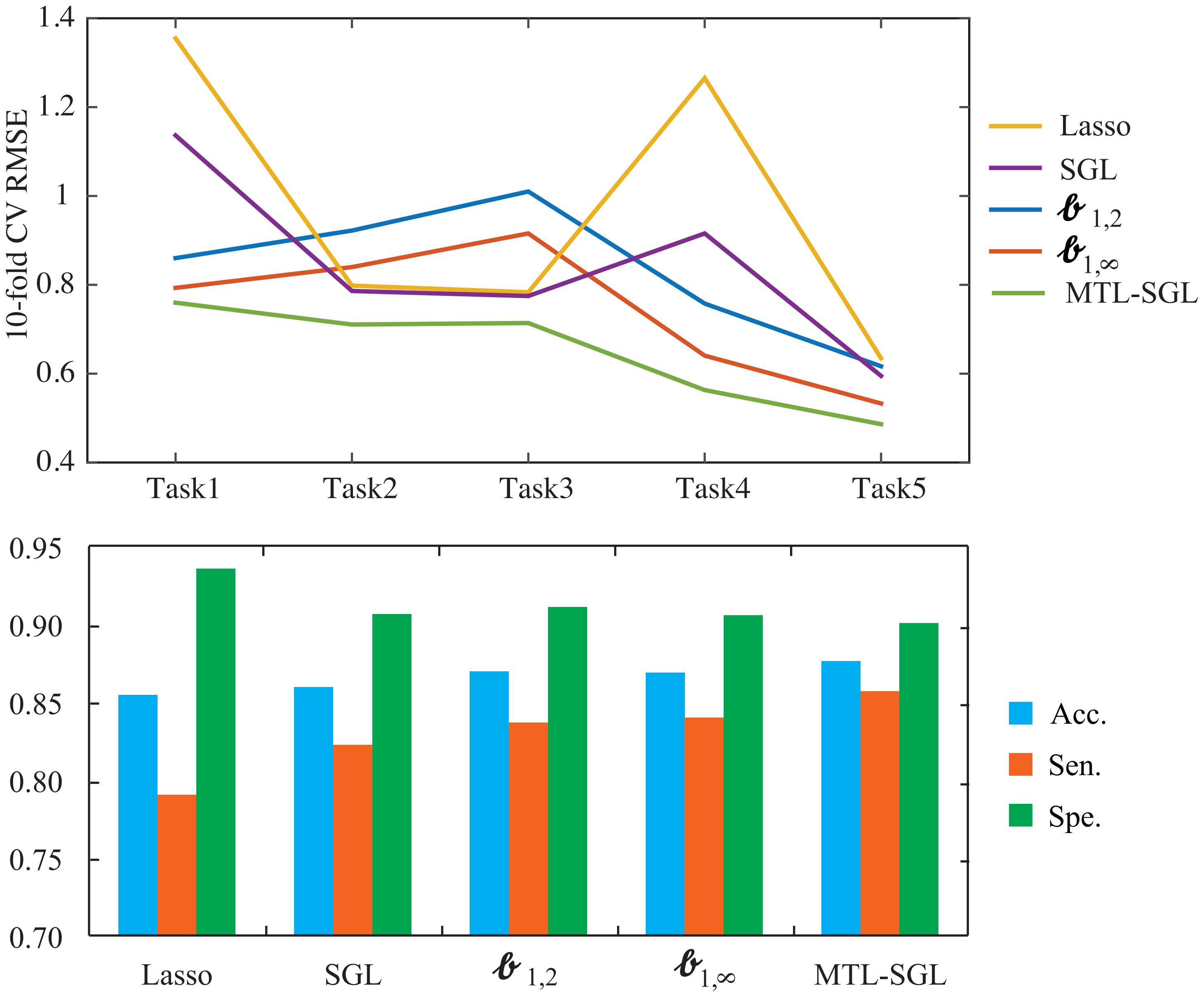

1.4
1.2
1
0.8
0.6
0.4
10-fold CV RMSE
Task1
Task2
Task3
Task4
Task5
Lasso
SGL
$\ell_{1,2}$
$\ell_{1,\infty}$
MTL-SGL
0.95
0.90
0.85
0.80
0.75
0.70
Acc.
Sen.
Spe.
Lasso
SGL
$\ell_{1,2}$
$\ell_{1,\infty}$
MTL-SGL

Figure S3

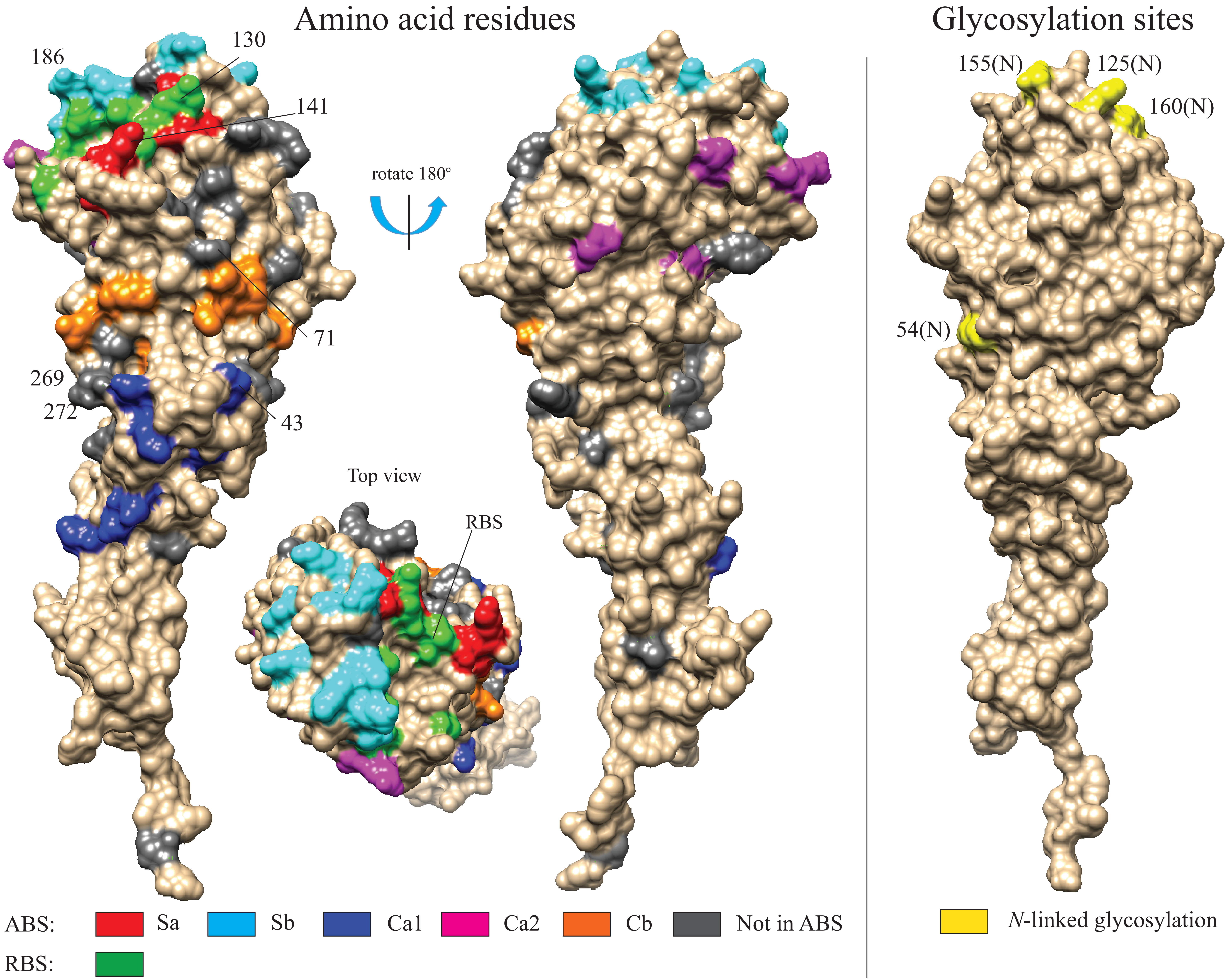

Amino acid residues
186
130
141
71
269
272
43
rotate 180°
Top view
RBS
Glycosylation sites
155(N)
125(N)
160(N)
54(N)
ABS:
Sa
Sb
Ca1
Ca2
Cb
Not in ABS
RBS:
N-linked glycosylation

Figure S4

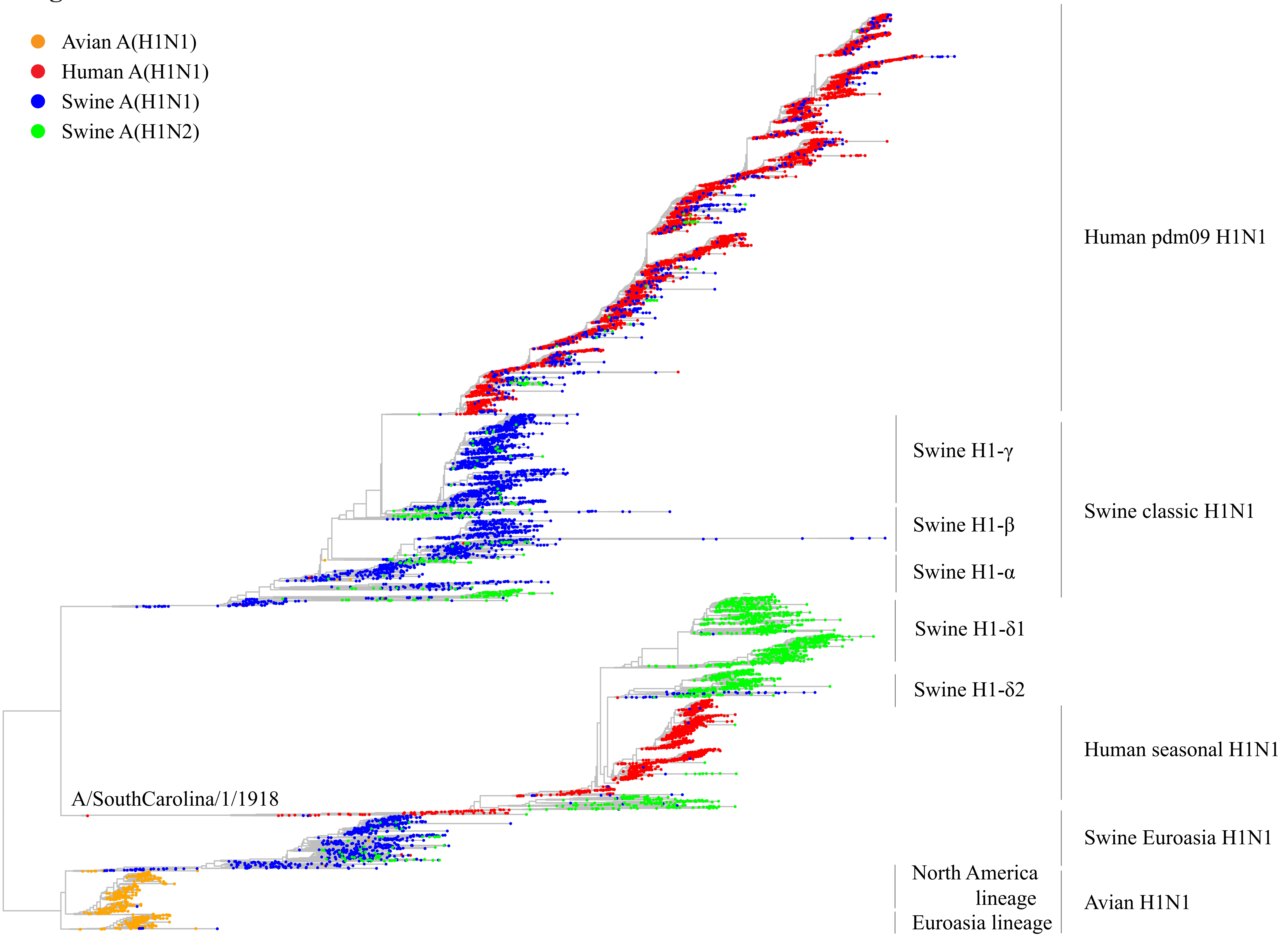

Avian A(H1N1)
Human A(H1N1)
Swine A(H1N1)
Swine A(H1N2)
Human pdm09 H1N1
Swine H1-γ
Swine H1-β
Swine H1-α
Swine classic H1N1
Swine H1-δ1
Swine H1-δ2
Human seasonal H1N1
A/SouthCarolina/1/1918
Swine Euroasia H1N1
North America lineage
Euroasia lineage
Avian H1N1

Figure S5

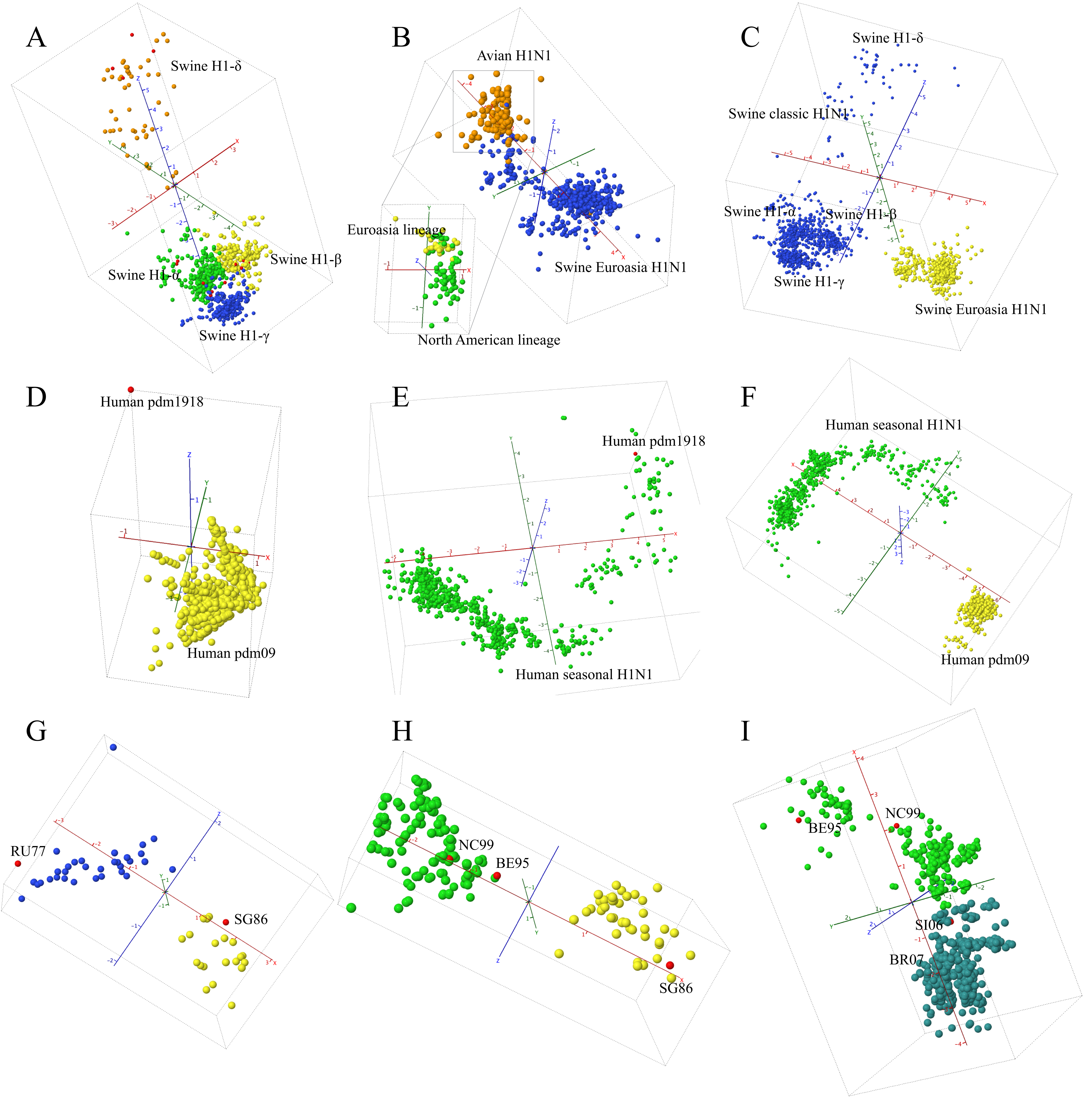
A
Swine H1-δ
Swine H1-β
Swine H1-α
Swine H1-γ
B
Avian H1N1
Euroasia lineage
Swine Euroasia H1N1
North American lineage
C
Swine H1-δ
Swine classic H1N1
Swine H1-α
Swine H1-β
Swine H1-γ
Swine Euroasia H1N1
D
Human pdm1918
Human pdm09
E
Human pdm1918
Human seasonal H1N1
F
Human seasonal H1N1
Human pdm09
G
RU77
SG86
H
NC99
BE95
SG86
I
NC99
BE95
SI06
BR07
